# Influences of luminance contrast and ambient lighting on visual context learning and retrieval

**DOI:** 10.3758/s13414-020-02106-y

**Published:** 2020-09-04

**Authors:** Xuelian Zang, Lingyun Huang, Xiuna Zhu, Hermann J. Müller, Zhuanghua Shi

**Affiliations:** 1 Institutes of Psychological Sciences, College of Education, Hangzhou Normal University, Hangzhou, 311121 People’s Republic of China; 2 Center for Cognition and Brain Disorders, Affiliated Hospital of Hangzhou Normal University, Hangzhou, Zhejiang Province 310015 People’s Republic of China; 3 Department of Educational and Counselling Psychology, McGill University, Montreal, Quebec Canada; 4 General and Experimental Psychology, Department of Psychology, Ludwig-Maximilians-Universität München, 80802 Munich, Germany

**Keywords:** Contextual cueing, Photopic vision, Mesopic vision, Contextual learning and retrieval

## Abstract

Invariant spatial context can guide attention and facilitate visual search, an effect referred to as “contextual cueing.” Most previous studies on contextual cueing were conducted under conditions of photopic vision and high search item to background luminance contrast, leaving open the question whether the learning and/or retrieval of context cues depends on luminance contrast and ambient lighting. Given this, we conducted three experiments (each contains two subexperiments) to compare contextual cueing under different combinations of luminance contrast (high/low) and ambient lighting (photopic/mesopic). With high-contrast displays, we found robust contextual cueing in both photopic and mesopic environments, but the acquired contextual cueing could not be transferred when the display contrast changed from high to low in the photopic environment. By contrast, with low-contrast displays, contextual facilitation manifested only in mesopic vision, and the acquired cues remained effective following a switch to high-contrast displays. This pattern suggests that, with low display contrast, contextual cueing benefited from a more global search mode, aided by the activation of the peripheral rod system in mesopic vision, but was impeded by a more local, fovea-centered search mode in photopic vision.

The visual system constantly encounters an overwhelming amount of information. To deal with this load, the system structures the information available in the environment and extracts statistical regularities to guide the allocation of focal attention. For example, people are quite proficient at using statistical regularities in a scene to detect and localize “target” objects, such as pedestrians appearing on the sidewalk and cars on the road, requiring particular re-/actions (Wolfe, Võ, Evans, & Greene, 2011). Visual search studies have also provided robust evidence that invariant spatial target–distractor relations can be extracted even from relatively abstract displays (rather than meaningful, real-life scenes) and be encoded in long-term memory (Chun & Nakayama, 2000; Goujon, Didierjean, & Thorpe, 2015), expediting search when the learnt layout is reencountered (Chun & Jiang, 1998, 1999; Wolfe & Horowitz, 2017)—an effect known as “contextual cueing.” In a typical contextual-cueing paradigm, observers search for a *T*-shaped target item amongst *L*-shaped distractors. Unbeknown to the observers, some of the search displays are repeatedly presented with the items arranged in an invariant spatial layout. Results show that observers can capitalize on those “hidden” regularities to facilitate search, compared with novel item arrangements (for a review see Chun, 2000).

Over the past 2 decades, researchers have identified factors mediating contextual cueing (Goujon et al., 2015). In particular, both the acquisition and the retrieval of spatial target–distractor associations critically depend on local inter-item relations available within an eye fixation (Geringswald & Pollmann, 2015; Zang, Jia, Müller, & Shi, 2015). For example, contextual cueing was effectively abolished when observers were provided with only two to three items near fixation by means of a gaze-contingent display manipulation (Zang et al., 2015). Contextual cueing is also very sensitive to target relocation (Makovski & Jiang, 2010; Zellin, Conci, von Mühlenen, & Müller, 2013) and the introduction of spatially irrelevant material between the target and the predictive context (Conci, Müller, & von Mühlenen, 2013; Olson & Chun, 2002). For example, Zellin et al. (2013) found that relocation of the target within a successfully acquired context effectively abolishes the cueing effect, even though the spatial distractor layout remains the same, because attention continues to be misguided to the old target location now occupied by a distractor (Manginelli & Pollmann, 2009; Zinchenko, Conci, Töllner, Müller, & Geyer, in press). And relearning of the new target location within the old distractor context requires extensive retraining (Zellin, von Mühlenen, Müller, & Conci, 2014). Collectively, these findings indicate that contextual cueing tolerates only limited changes of the context—in particular, changes affecting the local target–distractor relations. In the real world, though, such changes may be relatively rare (e.g., when we have misplaced some searched-for item, such as the kettle, from its usual position within a regular, kitchen-scene layout).

Another form of change, however, occurs quite frequently: Natural or artificial variations in ambient lighting can dramatically affect visibility and figure–ground contrasts in familiar contexts. For instance, the same street scene would appear very different between day and night conditions (e.g., a black car has a high contrast in daylight, but would turn near invisible at night). Thus, while spatial contexts in real environments are greatly affected by changes in ambient lighting and luminance contrast, the impact of these changes on contextual cueing has, to our knowledge, never been systematically examined. Rather, the acquisition of spatial context cues has been examined almost exclusively in relatively constant, standard laboratory lighting conditions (i.e., high stimulus-to-background contrast, “photopic” lighting above the mesopic level)—the implicit assumption being that the contextual-learning effects demonstrated under these conditions generalize across—that is, are independent of— variations in scene lighting and contrast factors. Clearly, however, this independence assumption—and, thus, the robustness of visual context learning and memory—must be verified empirically. In fact, there are reasons to doubt the validity of this assumption given that, for instance, changes in ambient lighting between day and night bring different visual-sensory systems into play (e.g., photopic and mesopic/scotopic vision), potentially affecting the extraction and thus the learning of structural relations and/or the recall of successfully acquired contextual regularities. Note that establishing in-/dependence of contextual cueing from variable environmental conditions would be not only of theoretical importance but also potentially of relevance for contextual training in certain real-world scenarios, such as driving simulation. On these grounds, the present study was designed to examine whether and how long-term statistical learning of spatial regularities, or “context cues,” in our visual environment depends on the prevailing ambient-lighting and luminance-contrast conditions, and whether and to what extent contextual cues acquired under certain lighting and contrast conditions could be effectively transferred to (i.e., be retrieved and continue to guide search under) changed environmental conditions.

Before elaborating the precise hypotheses tested in our study, it is useful to provide a brief review of the differential impacts of photopic (daylight) and mesopic/scotopic (low lighting/nightlight) vision on the “visual span” (Hulleman & Olivers, 2017; Legge, Ahn, Klitz, & Luebker, 1997; McConkie & Rayner, 1976)—that is, the area of the scene from which we can effectively take up information.

## Ambient lighting, stimulus contrast, and visual search

Ambient light intensity changes substantially from day to night. Our eyes adapt to this broad luminance range by changing the pupil size and switching between the photopic and mesopic/scotopic systems. Most of us have experienced the transition between the two systems when entering a dark place, such as the cinema, from bright daylight outside or when coming out of it. The underlying cause of this is that our retina is composed of rod and cone receptors that operate differently in bright and dark environments (e.g., Pokorny & Smith, 1997; Zele & Cao, 2015). Cones, which have their highest concentration in the fovea centralis, are responsible for color vision and function best in a bright, photopic environment. Rods, by contrast, are denser in the extrafoveal parts of the retina and support peripheral vision; they are entirely responsible for scotopic vision (Várady & Bodrogi, 2006; Zele & Cao, 2015), at luminance levels below 10^−3^ cd/m^2^, such as in a moonless night. Between photopic and scotopic vision, there is a transitional range of luminance from about 10^−3^ to 3 cd/m^2^, known as mesopic vision, in which both cones and rods contribute to the visual response (Pokorny & Smith, 1997; Zele, Maynard, & Feigl, 2013).

The shift of spectral sensitivity from photopic to mesopic-scotopic vision alters information processing in visual discrimination and identification tasks, given that less foveal and relatively more peripheral information is available in mesopic-scotopic vision (Pokorny & Smith, 1997; Zele & Cao, 2015). In addition, we face considerable changes and deficits in our perceptual ability (e.g., the Purkinje shift of the peak luminance sensitivity toward the blue end of the color spectrum; see Barlow, 1957). While performance degrades with increasing eccentricity in both photopic (Lee, Legge, & Ortiz, 2003) and mesopic vision (Paulun, Schütz, Michel, Geisler, & Gegenfurtner, 2015), the degradation is much weaker in the mesopic range. For instance, target detection is relatively unaffected by varying target eccentricity under mesopic vision conditions (Hunter, Godde, & Olk, 2017); but search for, say, a Gabor patch appearing in a peripheral region (e.g., at an eccentricity of 7.5° of visual angle) within a noisy background requires fewer saccades and is more efficient under scotopic compared with photopic conditions (Paulun et al., 2015). Findings such as these suggest that the visual system extracts useful information from a larger region of the visual field during each eye fixation (i.e., extending the visual span; Rayner, 1998) to compensate for the degradation of visual information in mesopic-scotopic vision, as compared with photopic vision.

In addition to luminance, stimulus contrast also affects visual processing. In a typical photopic search scenario (Greene, Brown, & Paradis, 2013; Näsänen, Ojanpää, & Kojo, 2001), the average response time and the number of fixations per search decrease with increasing display contrast. A similar relation has been found in the mesopic and scotopic ranges (Walkey, Harlow, & Barbur, 2006): Reaction time decreases exponentially with increasing luminance contrast. However, as shown by Paulun et al. (2015), detection sensitivity is shifted toward the periphery in scotopic lighting—that is, sensitivity for low-contrast Gabor patches was lowest at the center of the visual field, then gradually increased towards about 5° eccentricity in the periphery, and remained relatively constant between 5° and 15°; in photopic vision, by contrast, detection sensitivity reduced greatly in the peripheral vision. Taken together, these studies suggest that the visual system adjusts the visual span and discrimination sensitivity differentially between photopic and mesopic vision.

## Contextual cueing and central and peripheral vision

While light intensity and display contrast greatly influence visual search, it is unclear whether statistical learning of spatial target–distractor regularities within the search arrays would work in the same way under different luminance and item-to-background contrast conditions. Research on contextual learning and retrieval has revealed the availability of invariant local context relations within the viewing span to be crucial for contextual cueing. For instance, Geringswald, Baumgartner, and Pollmann (2012) observed that the loss of central-foveal vision (by computer simulation) in visual search eliminates contextual cueing. A further study of a group of participants with age-related macular degeneration (AMD), who suffer from impaired foveal vision, showed that they profit less from contextual cues compared with a control group of unimpaired observers (Geringswald, Herbik, Hoffmann, & Pollmann, 2013). Similarly, when the viewing span was limited (e.g., two to three items) within each fixation by means of a gaze-contingent display manipulation, Zang et al. (2015) also found barely any effect of contextual cueing. But when the whole display was made available unrestricted, contextual cueing manifested immediately—indicating that limiting the visual span effectively blocks the retrieval of already learnt contexts. Moreover, when the whole spatial configuration of the search items (but not their identity) was briefly presented (for 150 ms) prior to gaze-contingent search, the limited local invariant context was able to facilitate search—indicating that a certain amount of global context is required for successful contextual retrieval. Thus, the extant studies using gaze-contingent viewing manipulations and, respectively, AMD patients point to separate roles of local and global spatial-relational information for contextual cueing, and studies with gaze-contingent viewing manipulations reveal differential contributions of foveal and peripheral information to the cueing effect.

Differential contributions of the global and local context have also been confirmed in studies of search in naturalistic scenes (Brockmole, Castelhano, & Henderson, 2006; Brooks, Rasmussen, & Hollingworth, 2010). Brockmole et al. (2006) devised a local context (e.g., a table) containing a search target (a letter) embedded in a global context (e.g., a library scene). Their findings revealed contextual cueing to be biased towards global-context associations: In a transfer block (following initial learning), when the target appeared at the same location within the same global context (e.g., the library scene), contextual cueing was preserved even when the local context was changed (e.g., changing the table); but changing the global context abolished contextual cueing. Varying the association of a global scene with a local search array, Brooks et al. (2010) further demonstrated that, under certain conditions, the global–local context representations may organized hierarchically in determining contextual learning and retrieval: When a (predictive) global scene was uniquely associated with a local (repeated) search array, changing the global scene disrupted contextual cueing—consistent with Brockmole et al. (2006). However, no nesting of the local within the global representation was evident when a (repeated) search array was not consistently paired with a global scene during initial learning; in this case, contextual cueing remained robust despite changes of the global background.

Collectively, these studies—whether using just an abstract search array or a more scene-based search scenario—point to the important roles of local and global context in learning and retrieval of context cues. However, all of these studies were conducted under photopic, high-contrast lighting and stimulus conditions, so it remains unclear whether the changes of the visual span and sensitivity brought about by switching between photopic and mesopic vision (see above) would exert the same influences on contextual cueing. In the present study, we investigated the role of these factors in a “traditional” contextual-cueing paradigm with meaningless, artificial stimuli, which afford greater control of display variables compared with naturalistic scenes (where lighting sources, surface reflectances, etc., would need to be taken into account). This is not to say that the findings necessarily extend one-to-one to naturalistic scenes, which, qua being meaningful, provide additional cues deriving from “scene grammar” (e.g., Võ & Wolfe, 2013; Wolfe et al., 2011). However, it is reasonable to assume that changes of the lighting conditions engender similar adjustments of basic visual information processing (balance of rod/cone system, size of visual span), regardless of whether the scene is artificial or naturalistic. (For a more detailed consideration of the issue of generalizability, see the General Discussion.)

## Overview of the present study

Based on the findings reviewed above, we expected display contrast and environmental lighting to influence the size of the visual span and thereby impact spatial context learning and the retrieval of (acquired) contextual cues. Of note, we examined not only for contextual learning under certain lighting and stimulus contrast conditions but also for the transfer of any acquired cueing effects from the (initial) learning to a (subsequent) test session with (in some experiments) unchanged and (in others) changed lighting and contrast conditions. We specifically hypothesized that, compared with high-contrast stimuli, low-contrast stimuli presented under conditions of photopic vision would narrow the visual span, thus hampering contextual learning and/or retrieval. By contrast, under mesopic conditions, discrimination sensitivity for low-contrast displays would be boosted by activation of the rod system, expanding the visual span and thus facilitating contextual cueing of low-contrast contexts compared with photopic vision. In addition, transition from high-luminance (during contextual learning) to low-luminance contrast (during testing) or from mesopic to photopic vision with low-luminance contrast may narrow the visual span, hampering contextual retrieval.

**Fig. 1:**
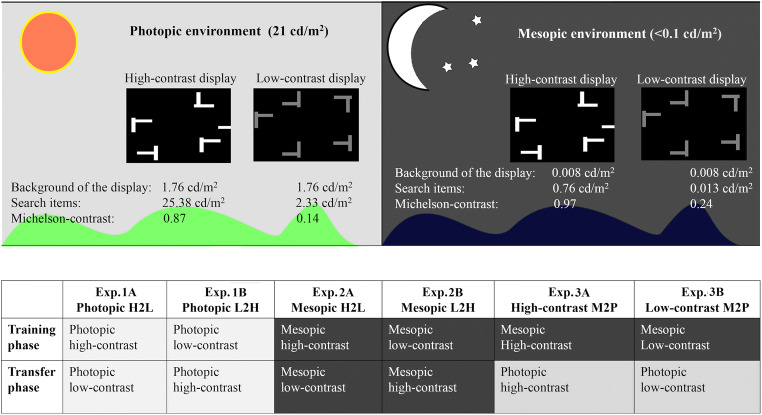
Overview of the design of the present study (of three experiments), along with illustrations of the search displays in the training and transfer sessions, and the luminance contrast and ambient-light conditions in the individual experiments. Lighter backgrounds indicate photopic environments (P); darker backgrounds indicate mesopic environments (M). H denotes high-contrast displays, L denotes low-contrast displays. H2L means the transition from high contrast in the training session to the low contrast in the transfer session; L2H from the low-contrast training to the high-contrast transfer; M2P from the mesopic training to the photopic transfer

To test these hypotheses, we designed three experiments (each consisting of two subexperiments, “A” and “B”) with high-contrast and, respectively, low-contrast search displays presented in photopic and mesopic environments, to examine for differential contextual-learning effects (in the initial training sessions) and transfer effects (in the subsequent transfer sessions). In particular, as almost all published contextual-learning studies have been conducted in photopic vision, we focused on contextual learning under mesopic vision conditions. Figure 1 illustrates the design of the present study.

Participants were instructed to search for, and respond to, a *T*-shaped target presented amongst *L*-shaped distractors. To examine for contextual learning and transfer effects across high/low stimulus contrasts and photopic/mesopic lighting environments, each experiment was divided into an initial training phase followed by a transfer phase. During training, the luminance contrast was set to high in Experiments 1A, 2A, and 3A, and to low in Experiments 1B, 2B, and 3B; in the transfer phases, the luminance contrasts were swapped in Experiments 1–2, but remained the same as in the training phases in Experiment 3 (see Fig. 1). Moreover, the ambient environment was kept the same for Experiments 1 and 2 (photopic for Experiment 1, mesopic for Experiment 2), but was switched from mesopic during training to photopic in the transfer phase for Experiment 3.

To preview the results, there was successful contextual learning with both high-contrast and low-contrast displays in mesopic vision, but in photopic vision, only with high-contrast and not low-contrast displays. Moreover, there was an effective transfer of contextual cueing from low-contrast to high-contrast displays, but not vice versa. In addition, switching lighting from mesopic to photopic vision caused the contextual-cueing effect associated with low-contrast displays to be diminished. Overall, these findings are consistent with the idea that the ambient-lighting and stimulus-contrast conditions are major modulators of contextual learning and retrieval, and work by influencing the effective perceptual span during visual scanning (see General Discussion).

## Experiment 1

Experiment 1 was designed to investigate the influence of item-background contrast on contextual learning and transfer under photopic vision conditions. To this end, the display contrast was set to high in the training session but to low in the subsequent transfer session in Experiment 1A. Conversely, in Experiment 1B, the display contrast was set to low in the training session, but set to high in the transfer session (see Fig. 1).

### Method

#### Participants

Two separate groups, of 30 participants each, took part in Experiments 1A and 1B (18 females, mean ages = 25.2 years and 25.4 years), respectively. All participants had normal or corrected-to-normal visual acuity. The sample size was estimated by a power analysis using G*Power (Prajapati, Dunne, & Armstrong, 2010). In a standard contextual-cueing task, the effect size is relatively large (e.g., *f* > .65 in Zang, Shi, Müller, & Conci, 2017). Here, we used *f* = .65 in the estimation, which yielded a sample size of 28 per experiment to reach a power of 90% and an α level of .05. To be more conservative, we recruited 30 participants for each experiment.

All participants gave written informed consent prior to the experiment and were paid for their participation. The study was approved by the ethics committee of the Ludwig Maximilian University of Munich (LMU) Psychology Department in accordance with the Declaration of Helsinki, and the procedures were carried out in accordance with the relevant guidelines and regulations. (This also applies to Experiments 2 and 3, reported below.)

#### Apparatus

The experiment was conducted in a dimly lit experimental cabin, with stimuli presented on a 21-inch LACIE CRT monitor (screen resolution: 1,024 × 768 pixels; refresh rate: 100 Hz). The monitor brightness and contrast was set to 50%, and the cabin was lit by a ceiling lamp to a photopic level of environmental lighting (21 cd/m^2^). The viewing distance was fixed at 57 cm, maintained with the support of a chin rest. Stimulus presentation was controlled by using Psychtoolbox (Brainard, 1997) and MATLAB codes.

#### Stimuli

The search stimuli consisted of one *T*-shaped target rotated 90° or 270° from the vertical (i.e., the *T* was oriented in either rightward-pointing or leftward-pointing direction) and 15 *L*-shaped distractors (randomly rotated by 0°, 90°, 180° or 270°). Similar to previous studies (Jiang & Chun, 2001; Zang et al., 2015), the *L* distractors had a small offset (0.15°) at the line junctions, making the *L*s more similar to the target *T*. Each stimulus subtended 1.0° of visual angle. All search items were randomly presented on four concentric (invisible) circles with radii of 2°, 4°, 6°, and 8° of visual angle, respectively. Targets appeared only on the second or the third circle, while distractors could appear on all the four circles; this item arrangement is identical to that used in previous contextual-cueing studies (Annac et al., 2013).

As illustrated in Fig. 1, the search items (*T* and *L*s) were presented on a dark-gray background (1.76 cd/m^2^). In Experiment 1A, the luminance of the search items was set to high (25.38 cd/m^2^) during the training session, and to low (2.33 cd/m^2^) during the transfer session. Conversely, in Experiment 1B, search-item luminance was low (2.33 cd/m^2^) during the training session, but high (25.38 cd/m^2^) during the transfer session. We calculated the display contrast (0.87 and 0.14 of the high-contrast and low-contrast displays, respectively) in terms of the Michelson contrast, which is defined as(*I*_*i*_ − *I*_*b*_)/(*I*_*i*_ + *I*_*b*_), where *I*_*i*_ and *I*_*b*_ represent the luminance of the search items and the background, respectively.

#### Procedure

Both experiments consisted of a training session of 25 blocks, followed by a transfer session of five blocks and a recognition test. Each block consisted of 16 trials, eight with “old” and eight with “new” displays, presented in randomized order. For the old displays, the locations of all the search items (both the *T* and the *L*s) were kept constant and repeated once per block during the experiments; for the new displays, by contrast, the locations and orientations of the distractors were randomly determined for each presentation. To maintain comparable repetitions of the target locations for both old and new displays, targets also appeared at eight predefined locations in the new displays. The orientation of the target (leftward pointing vs. rightward pointing) was randomly selected for each search display, whether new or old, thus preventing any RT advantage from constant target orientation in old versus randomly variable orientation in new displays.

Participants were instructed to find the target *T* amongst the distractors *L*s and discern its orientation (leftward pointing or rightward pointing), as rapidly and accurately as possible, by pressing either the left or the right arrow key on the keyboard with their left or right index finger, respectively. Each trial started with the presentation of a central fixation cross for 800–1,000 ms, followed by a search display that remained on the screen until a response was made or until the presentation exceeded 10 seconds. The next trial started after a random intertrial interval (ITI) of 1.0–1.2 seconds.

Prior to performing the visual search task, participants practiced the task in one block of 16 trials, in which the luminance contrast of the stimuli and the ambient lighting were set to the more challenging settings used in the training or the test session (i.e., low contrast in both Experiment 1A and 1B). This was done to ensure that participants were able to actually perform the task under the more difficult conditions—the assumption being that if participants reached an accuracy greater than 75% in the more difficult practice condition, they would also be able to perform the task under the easier condition. Participants who performed worse than 75% correct practiced the task again for two or three blocks, until they reached the accuracy criterion. No participants were excluded based on the current criteria. The item configurations displayed in the practice session were not used in the later parts of the experiment. Participants were free to take a break between blocks.

After the formal visual search experiment (i.e., training and test sessions), participants received one block of the recognition test, consisting of eight old-display and eight new-display trials. Participants had to make two-alternative forced choices as to whether a given display was an “old” or a “new” one by pressing the left or the right arrow key, respectively. The ambient lighting condition and the display contrast were the same as was used during the training sessions. The same dark-adaptation procedure was adopted for the recognition test in the mesopic environment. Of note, participants were explicitly informed before the recognition test that half of the recognition displays were old while the other half were new.

#### Statistical analyses

Statistical testing was mainly based on analyses of variance (ANOVAs). To establish whether critical nonsignificant effects favor the null hypothesis, we calculated Bayes factors (*BF*) with JASP (0.11.1), using the default Cauchy settings (i.e., *r*-scale fixed effects = 0.5, *r*-scale random effects = 1, *r*-scale covariates = 0.354). Likewise, for Bayesian *t* tests, we used the default Cauchy prior (scale of 0.707). All Bayes factors reported for ANOVA main effects and interactions are “inclusion” Bayes factors calculated across matched models. Inclusion Bayes factors compare models with a particular predictor to models that exclude that predictor. That is, they indicate the amount of change from prior inclusion odds (i.e., the ratio between the total prior probability for models including a predictor and the prior probability for models that do not include it) to posterior inclusion odds. Using inclusion Bayes factors calculated across matched models means that models that contain higher-order interactions involving the predictor of interest were excluded from the set of models on which the total prior and posterior odds were based. Inclusion Bayes factors provide a measure of the extent to which the data support inclusion of a factor in the model. *BF* values less than 0.33 are taken to provide substantial evidence for the null hypothesis (Kass & Raftery, 1995).

### Results

#### Error rates

**Fig. 2:**
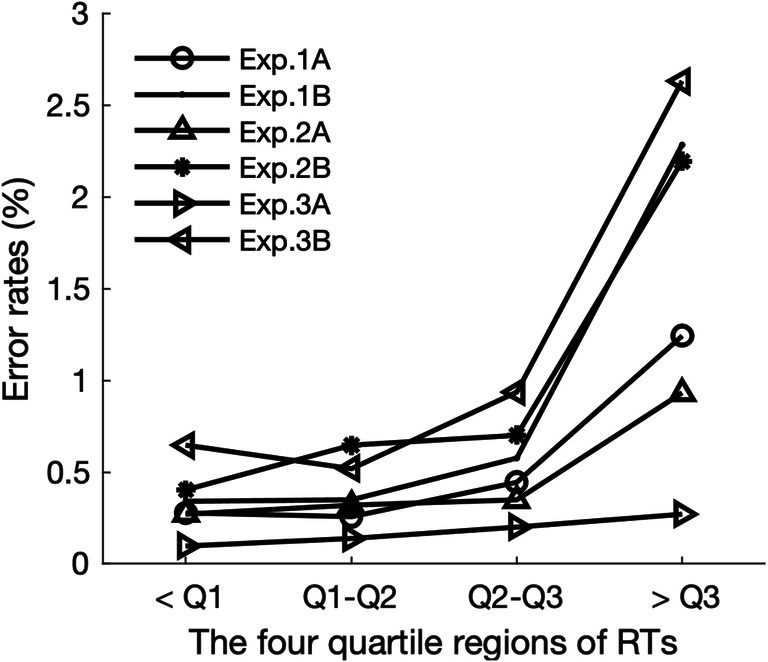
Mean error rates as a function of RT quartile subset, for each experiment. Q1, Q2, and Q3 denote the 25%, 50%, and 75% quartiles, respectively

The proportion of trials with response errors and response failures (trials without response within the allowed time) were low (Experiment 1A, errors: 2.22%, failures: 0.85%; Experiment 1B, errors: 3.55%, failures: 1.69%). To examine for potential speed–accuracy trade-offs, we grouped the trials into four quartile subsets according to the response times (RTs) and examined the respective (quartile-subset) error rates. This analysis revealed that most errors were made with the slowest responses (see Fig. 2). One-way repeated-measures ANOVAs confirmed this RT quartile-subset effect to be significant for each experiment, Experiment 1A, *F*(3, 87) = 23.79, *p* < .001, η_p_^2^ = .45; Experiment 1B, *F*(3, 87) *=* 34.80, *p* < .001, η_p_^2^ = .55. This effectively rules out a trade-off between the accuracy and speed of responses. Accordingly, trials with response errors and failures to respond were excluded from further RT analysis.

#### Contextual-cueing effects

**Fig. 3:**
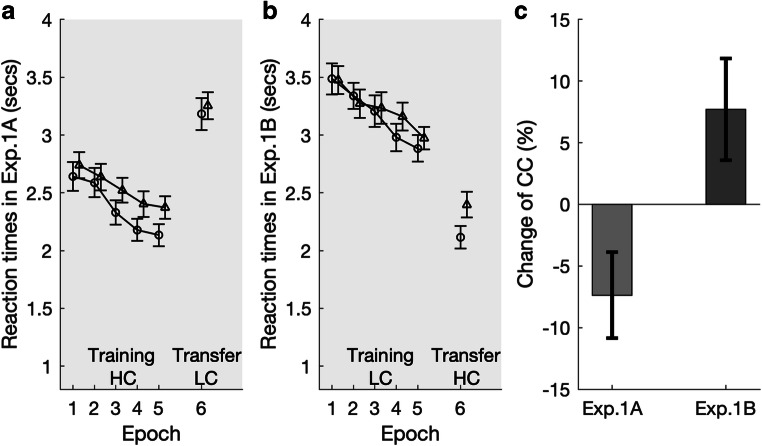
Results of Experiment 1. **a–b** Mean RTs, with associated standard errors, for the old (open circles) and new contexts (open triangles) as a function of task epoch. Light background shading indicates that the experiment was conducted under photopic vision. In Experiment 1A, stimulus contrast was high (HC) in the training session, but low (LC) in the transfer session; this was reversed in Experiment 1B. **c** Percentage change (negative: decrease; positive: increase) of the contextual-cueing (CC) effect from the last epoch of the training session to the test session for Experiments 1A–B. The change was significant for Experiment 1A (*p* = .043), but only marginal significant for Experiment 1B (*p* = .07)

For the RT analysis, we grouped every five consecutive trial blocks (of 16 trials each) into an “epoch” (of 80 trials), yielding five task epochs for the training session and one epoch for the transfer session. Figures 3a–b show the mean RT as a function of the epoch and display context. To examine the contextual-cueing effects, for each experiment, RT performance in the training session was subjected to a repeated-measures ANOVA with the factors context (old vs. new) and epoch (1 to 5), and RT performance in the transfer session by an ANOVA with the single factor context.

Using a standard setting (i.e., high item-background contrast in the photopic environment), the training session of Experiment 1A replicated the standard contextual cueing effect, *F*(1, 29) = 5.81, *p* = .022, η_p_^2^ = .17, with an overall RT facilitation of 160 ms for the old versus new displays. The effect of epoch was also significant, *F*(4, 116) = 22.07, *p* < .001, η_p_^2^ = .43: response speed increased across the training session (436 ms faster RTs in Epoch 5 vs. Epoch 1), indicative of procedural learning (i.e., general learning of how to perform the task). The significant Context × Epoch interaction, *F*(4, 116) = 2.54, *p* = .044, η_p_^2^ = .08, indicates that the contextual-cueing effect developed as the experiment progressed (see Fig. 3a).

For the subsequent transfer session of Experiment 1A, in which the item-background contrast was switched from high to low, a paired-sample *t* test, with context (old vs. new) as a factor, showed nonsignificant result, *t*(29) = 0.64, *p* = 0.53, Cohen’s *d* = 0.12, *BF*_*10*_ = 0.24, indicating reduced contextual cueing. Note that the mean RT was still 72 ms faster to old versus new configurations, yet due to large interparticipant variation, this difference was not significant (in fact, the Bayes factor favors the null hypothesis of no contextual cueing). To examine the transfer effect of contextual cueing, we further estimated the change of contextual cueing from the last block of the training session (i.e., Epoch 5) to the test session (i.e., Epoch 6) for each experiment. Given that the changes of the lighting and stimulus-contrast conditions between the training and test sessions had a substantial impact on general response speed, we calculated the transfer effect based on the relative contextual-cueing magnitudes (calculated by relating the mean contextual-cueing effect to the mean RT). Figure 3c depicts the percentage change of the normalized contextual-cueing effects (i.e., the percentage of the difference in RTs to new minus old displays related to RTs to new displays): 100 × (RT(new) − RT(old)) / RT(new)%, from the training to the test session. A simple *t* test revealed the reduction of the cueing effect (−7.36%) to be significant, *t*(29) = 2.11, *p* = .043. Together with the nonsignificant cueing effect in the test session (see above), this indicates that contextual cues extracted and learned from high-contrast displays (in the training session) could not be effectively transferred to low-contrast displays—that is, presenting search displays with low item-background contrast in daylight conditions impedes the expression of (acquired) contextual cueing.

By contrast, when presenting low-contrast displays under photopic conditions in the training session in Experiment 1B (see Fig. 3b), neither the main effect of context, *F*(1, 29) = 0.38, *p* = .54, η_p_^2^ = .01, *BF*_*incl*_ = 0.21, nor the Context × Epoch interaction, *F*(4, 116) = 1.41, *p* = .23, η_p_^2^ = .05, *BF*_*incl*_ = .088, turned out to be significant, with the Bayes factor favoring the null hypothesis of no contextual cueing during the training session (though RTs were 44 ms faster for old than for new displays). Only the main effect of epoch was significant, *F*(3.06, 88.65) = 22.02, *p* < .001, η_p_^2^ = .43, again reflecting significant procedural learning (552 ms shorter RTs in Epoch 5 compared with Epoch 1). Interestingly, however, while there was only numerical contextual facilitation in the (low-contrast) training session (44-ms effect, *p* = .54), a significant cueing effect emerged following the switch to high-contrast displays in the transfer session, *t*(29) = 2.84, *p* = .008, Cohen’s *d* = 0.52: There was an RT advantage of 280 ms for repeated versus nonrepeated displays. However, the change in the relative measure was only marginal (7.71%; see Fig. 3c), *t*(29) = 1.87, *p* = .07, *BF*_*10*_ = 0.904. Given that a substantial contextual-cueing effect was already evident in the first block of the transfer session (228, 238, 258, 420, and 265 ms for Blocks 1 to 5, respectively), the invariant spatial context was likely acquired in (and transferred from) the training session, rather than reflecting a relearning effect developed in the transfer session. However, the low-contrast setting in the training session may have limited the expression of contextual cueing.

### Discussion

Taken together, for conditions of photopic lighting, the findings of Experiment 1A revealed successful contextual learning under the high-contrast condition, but this acquired contextual facilitation could not be transferred to the low-contrast condition; by contrast, the findings of Experiment 1B suggest that repeated spatial arrangements could be successfully learned with low-contrast displays, but contextual facilitation was expressed only when the display contrast changed to high. The findings of Experiments 1A and 1B suggest that visual search displays encountered in daylight conditions at low item-to-background contrast impede contextual retrieval, but not contextual learning. Previous studies had shown that the visual span contracts with decreasing display contrast (Greene et al., 2013; Näsänen et al., 2001; Paulun et al., 2015). Accordingly, the impaired contextual retrieval observed here is likely attributable to the visual span being reduced under conditions of low display contrast. Given the likely extension of the visual span under conditions of mesopic-scotopic vision (Paulun et al., 2015), we went on to investigate the role of display contrast in mesopic vision in Experiment 2.

## Experiment 2

Experiment 2 was designed to examine the influence of display contrast on contextual cueing in mesopic vision. The experimental details were essentially the same as in Experiment 1, except that participants performed the tasks in a dark environment, with all light sources, apart from the display CRT, eliminated (environment illumination <0.1 cd/m^2^) and with appropriately adjusted screen-background and stimulus luminance settings.

### Method

#### Participants

Two groups of 30 participants completed Experiments 2A and 2B (18 and 14 females, mean ages of 25.3 and 29.4 years), respectively. Note that two participants had to be replaced in Experiment 2B because they failed to achieve the accuracy criterion on the practice trials (see Experiment 1, above).

#### Stimuli and procedure

To reduce the background lighting of the CRT screen to mesopic level, the monitor brightness was set to 0% and the contrast to 20% (0.008 cd/m^2^). Before the start of the experiments, participants were given 10 minutes for dark adaptation (i.e., for their mesopic/scotopic vision systems to become active). The item-to-background contrast was changed from high (0.76 cd/m^2^) in the training session to low (0.013 cd/m^2^) in the transfer session in Experiment 2A, and from low to high in Experiment 2B. The low-luminance contrast was calculated via multiple measures of luminance and curve fitting. This is because, under mesopic vision conditions, the luminance levels of the background and the search items were close to the lower measurable boundary of the Minolta Chroma Meter CS-100 (0.01 cd/m^2^). To reduce measurement noise, we took multiple measures and used curve fitting to obtain the luminance-characteristic curve of the LACIE CRT monitor from the RGB range between [255, 255, 255] to [80, 80, 80] (with display contrast set to 20% and brightness to 0%). Based on this, we extrapolated the luminance with RGB values of [30, 30, 30] (setting of the background) and of [35, 35, 35] (setting of the search items). This yielded estimated values of 0.008 cd/m^2^ and 0.013 cd/m^2^ for the background and the search items, respectively, for the low-contrast displays. After the dark adaptation, all participants reported that they could see the search items.

All other experimental details were the same as those in Experiment 1.

### Results

#### Error rates

Similar to Experiment 1, response-error rates (1.87% and 3.94% in Experiments 2A and 2B, respectively) and response-failure rates (0.35% and 1.79%, respectively) were low. Again, we grouped the trials into four quartile subsets according to the RTs and examined the (quartile-subset) error rates in one-way repeated-measures ANOVAs. The pattern was similar to Experiment 1 (see Fig. 2): There were significant RT quartile-subset effects—Experiment 2A, *F*(1.65, 47.86) = 10.11, *p* < .001, η_p_^2^ = .26; Experiment 2B, *F*(1.54, 44.77) = 30.35, *p* < .001, η_p_^2^ = .51—with error rates being increased for the slowest RTs, ruling out any trade-off between the accuracy and speed of responses. Accordingly, trials with response errors and failures to respond were excluded from further RT analysis.

#### Contextual-cueing effects

**Fig. 4:**
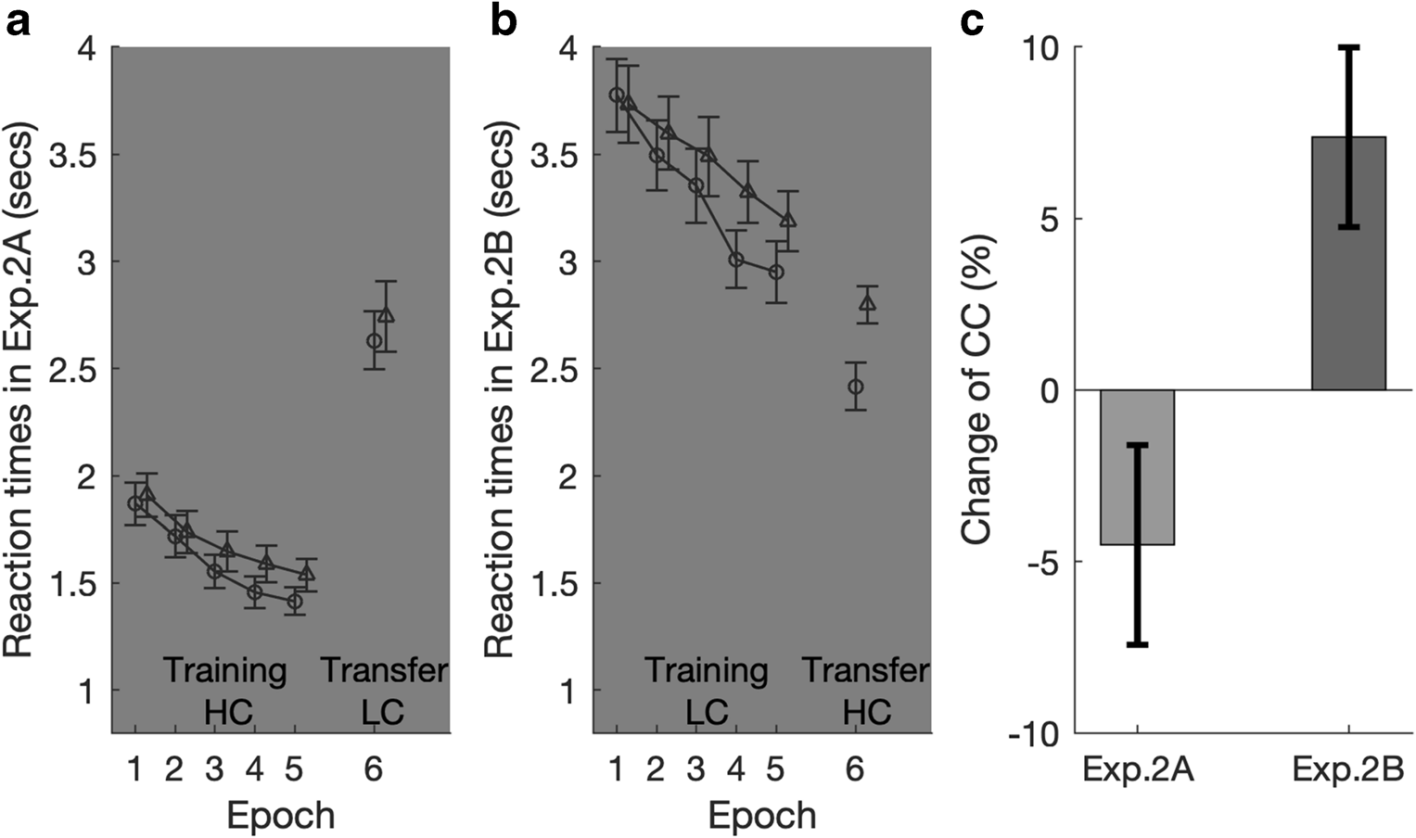
Results of Experiment 2. **a–b** Mean RTs, with associated standard errors, for the old (open circles) and new contexts (open triangles) as a function of task epoch. Dark background shading indicates that the experiment was conducted under mesopic vision. In Experiment 2A, stimulus contrast was high (HC) in the training session, but low (LC) in the transfer session; this was reversed in Experiment 2B. **c** Percentage change (negative: decrease; positive: increase) of the contextual-cueing (CC) effect from the last epoch of the training session to the test session for Experiments 2A–B

The contextual-cueing effects for Experiments 2A and 2B are depicted in Figs. 4a and 4b, respectively. The results of Experiment 2A (conducted in a mesopic environment) were generally similar to Experiment 1A (photopic environment). For the training session, both main effects were significant: epoch, *F*(2.08, 60.29) = 38.49, *p* <.001, η_p_^2^ = .57; 412 ms faster responding in Epoch 5 versus Epoch 1, and the context, *F*(1, 29) = 4.78, *p* = .037, η_p_^2^ = .14; 81-ms RT advantage for repeated versus nonrepeated displays. But the Context × Epoch interaction was not significant, *F*(3.20, 92.68) = 1.49, *p* = .21, η_p_^2^ = .05, *BF*_*incl*_ = .079. When the display contrast was switched to low in the transfer session, contextual facilitation was 110 ms numerically, but failed to reach significance, *t*(29) = 1.27, *p* = .21, Cohen’s *d* = 0.23, *BF*_*10*_ = 0.40; 2,630 versus 2,740 ms for repeated versus nonrepeated displays. Consistent with this pattern, the relative change of normalized contextual cueing between the training and the test session in Experiment 2A (see Fig. 4c) was nonsignificant (−4.52%), *t*(29) = −1.55, *p* = .13, *BF*_*10*_ = 0.59. That is, while contextual cueing differed little between the training and test sessions, the uncertainty engendered by the luminance reduction in the test session rendered the expression of contextual cueing somewhat noisy.

In Experiment 2B, participants also performed the task in the mesopic environment, but display contrast was switched from low during training to high during (transfer) test. Under these conditions, for the training session, the results were different to Experiment 1B (photopic environment, low contrast during training): Participants showed contextual cueing with low-contrast displays in a mesopic environment. Both main effects were significant: epoch, *F*(2.32, 67.37) = 15.87, *p* < .001, η_p_^2^ = .35 (686 ms faster RTs in Epoch 5 vs. Epoch 1), and context, *F*(1, 29) = 4.57, *p* = .04, η_p_^2^ = .14 (150-ms RT advantage for repeated vs. non-repeated displays), as well as the Context × Epoch interaction, *F*(3.18, 92.18) = 3.40, *p* = .01, η_p_^2^ = .11. Furthermore, this successfully established cueing effect was transferred to the test session, in which the display contrast was switched to high (mean RT facilitation of 380 ms)—as confirmed by an ANOVA, with context as the main factor for the test session: *F*(1, 29) = 20.86, *p* < .001, η_p_^2^ = .42, *t*(29) = 4.57, *p* < .001, Cohen’s *d* = 0.84. In fact, the switch from low to high stimulus contrast led to a significant increase of the relative cueing effect, by 7.38%, *t*(29) = 2.82, *p* = .008. Note that, with 423 ms, the cueing effect was already very substantial in the first block of the transfer session (across the five transfer blocks, facilitation was 423, 300, 460, 352, and 407 ms, respectively). Accordingly, the increase in the relative magnitude of contextual cueing indicates that high stimulus contrast promotes the expression (rather than just the acquisition) of contextual cueing.

### Discussion

Taken together, the results of Experiment 2 indicate that contextual cues can be successfully learned with both high-contrast and low-contrast displays under mesopic vision. However, switching stimulus contrast from high to low (as in Experiment 2A) makes the expression of contextual facilitation more variable, resulting in an overall large yet nonsignificant contextual-cueing effect in the test session. This pattern is similar to Experiment 1A, in which a change from high to low contrast also rendered a noisy expression of contextual cueing, under photopic vision conditions. Both experiments (1A and 2A) suggest that switching stimulus contrast from high to low brings about a system adaptation to the change that involves a narrowing of the visual span, which makes contextual retrieval (activation of learnt context cues by display contents) more noisy and impedes the expression of the contextual cueing. This is consistent with Zang et al.’s (2015) demonstration that, when uptake of information about spatial inter-item relations in peripheral vision is prevented (by means of an artificial restriction of the visual span), even acquired contextual representations are hard to retrieve, resulting in a failure to guide search.

Comparison of Experiment 1B (photopic low to high contrast) with 2B (mesopic low to high contrast) indicates that contextual facilitation was overall increased, by some 100 ms, in mesopic vision, as compared with photopic vision, for both the training (150 ms vs. 44 ms) and the transfer sessions (380 ms vs. 280 ms). As shown by previous studies, in mesopic/scotopic vision, detection sensitivity is shifted toward the periphery and the visual system extracts information from a wider region around the current fixation point (Hunter et al., 2017; Paulun et al., 2015). That is, the visual span is expanded by the (peripheral) rod system coming into play in mesopic vision, and this may play a critical role in contextual retrieval. Indeed, with low-contrast displays, contextual cueing was expressed only under conditions of mesopic (Experiment 2B), but not photopic (Experiment 1B), vision, likely owing to the expansion of the visual span with the aid of the peripheral rod system. When the display contrast was changed to high, both Experiments 1B and 2B revealed a contextual-cueing effect (of 280 ms and 380 ms, respectively), likely owing to an adaptive expansion of the visual span in response to the high item-to-background contrast. Thus, converging evidence suggests that contextual learning can take place under conditions of both mesopic and photopic vision, but effective retrieval of learned contexts depends on the degree to which peripheral information about spatial inter-item relations becomes available—conferring a general advantage on mesopic over photopic vision.

## Experiment 3

While Experiment 2 looked at the effects of display-contrast changes on contextual cueing in mesopic vision, Experiment 3 examined the transfer of contextual cueing from mesopic to photopic conditions while keeping the display contrast constant. Given that Experiment 1B had shown that low-contrast displays may limit the expression of contextual cueing, we were particularly interested in ascertaining whether low-contrast contexts learned under conditions of mesopic vision (as evidenced by Experiment 2B) would be available for contextual guidance under conditions of photopic vision.

### Method

#### Participants, stimuli, and procedure

Two groups of 30 participants took part in Experiments 3A and 3B (21 and 18 females, mean ages of 25.8 and 24.3 years), respectively.

The experimental paradigm was essentially the same as in Experiments 1 and 2, except for the following differences: In both experiments, display contrast was fixed for the training and test sessions (high contrast in Experiment 3A, low contrast in Experiment 3B), while the environmental lighting was changed from mesopic to photopic. Participants were initially given 10 minutes for dark adaptation (i.e., for their mesopic/scotopic vision systems to become active).

### Results

#### Error rates

Similarly, participants mean response errors and response failures were low (Experiment 3A, 0.71% and 0.21%, respectively; Experiment 3B, 4.74% and 2.32%, respectively). More errors were made with slower responses, as confirmed by one-way repeated-measures ANOVAs yielding significant main effects of RT quartile-subset: Experiment 3A, *F*(3, 87) = 3.57, *p* = .017, η_p_^2^ = .45; Experiment 3B, *F*(3, 87) = 23.79, *p* < .001, η_p_^2^ = .45, thus effectively ruling out speed–accuracy trade-offs.

#### Contextual-cueing effects

**Fig. 5:**
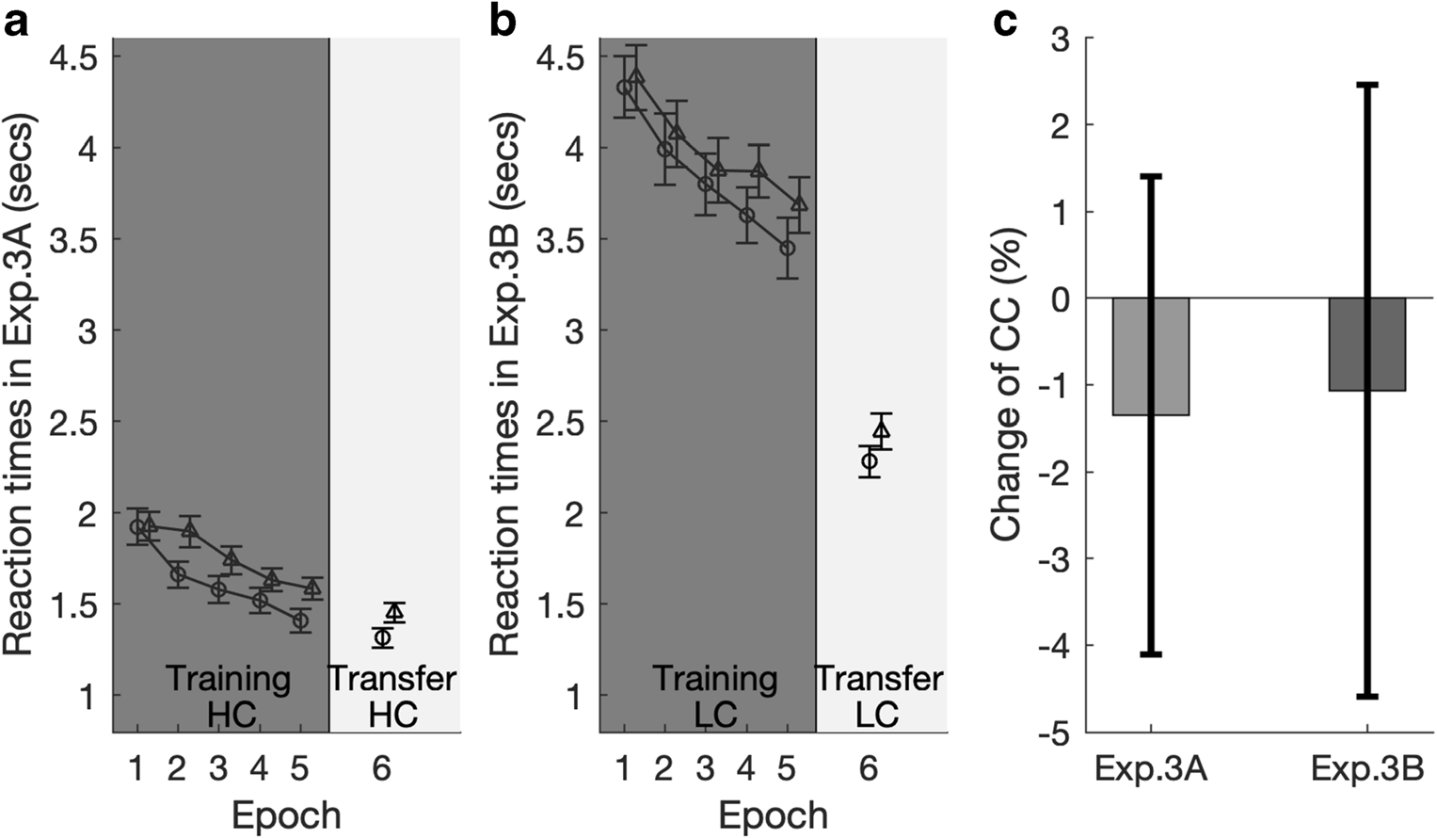
Results of Experiment 3. **a–b** Mean RTs, with associated standard errors, for the old (open circles) and new contexts (open triangles) as a function of task epoch. Dark background shading indicates that the search task was conducted in mesopic vision; light shading that it was conducted in photopic vision. Stimulus contrast was high (HC) in Experiment 3A, and low (LC) in Experiment 3B. **c** Percentage change (negative: decrease; positive: increase) of the contextual-cueing (CC) effect from the last epoch of the training session to the test session for Experiments 3A–B

The contextual-cueing effects for Experiments 3A and 3B are depicted in Figs. 5a and 5b, respectively. The (mesopic) training session of Experiment 3A replicated the results of the “equivalent” Experiment 2A (mesopic training with high-contrast displays)—all effects were significant: epoch, *F*(2.70, 78.41) = 43.61, *p* < .001, η_p_^2^ = .60 (431 ms faster RTs in Epoch 5 vs. Epoch 1); context, *F*(1, 29) = 8.74, *p* = .006, η_p_^2^ = .23 (mean facilitation effect of 138 ms); and Context × Epoch interaction, *F*(2.86, 82.83) = 4.46, *p* = .002, η_p_^2^ = .13. Importantly, the contextual-cueing effect was maintained—with an overall facilitation of 139 ms—in the transfer session in which the ambient lighting was switched from mesopic to photopic, *t*(29) = 3.24, *p* = . 003, Cohen’s *d* = 0.59. In fact, there was no evidence of a difference in the relative cueing effects between the training and test sessions (numerical difference of −1.35%; see Fig. 5), *t*(29) = 0.489, *p* = .63, *BF*_*10*_ = .22. In the test session, the mean facilitation effects were 117, 172, 128, 127, and 152 ms, respectively, in the five transfer blocks—likely reflecting successful transfer of the cueing effect from mesopic to photopic vision (rather than fast reacquisition under the latter condition).

Experiment 3B examined for contextual learning and transfer with low-contrast displays, with a change from a mesopic to a photopic environment. The results of the training session were similar to the “equivalent” Experiment 2B (mesopic training with low-contrast displays): apart from a significant effect of epoch, *F*(3.15, 91.23) = 23.29, *p* < .001, η_p_^2^ = .45 (790 ms faster RTs in Epoch 5 vs. Epoch 1), the main effect of context was significant, *F*(1, 29) = 4.73, *p* = .038, η_p_^2^ = .14, with an overall facilitation effect of 138 ms. The Context × Epoch interaction failed to reach significance, *F*(4, 116) = 1.56, *p* = .19, η_p_^2^ = .05, *BF*_*incl*_ = .052. When the ambient lighting was switched from mesopic to photopic, the contextual-cueing effect was only marginal, *t*(29) = 2.01, *p* = .054, *Cohen’s d* = 0.37, *BF*_*10*_ = 1.13, averaging 163 ms across the whole transfer epoch (facilitation of 296, 193, 104, 99, and 134 ms in Transfer Blocks 1 to 5, respectively)—though, measured in terms of the relative effect, it remained equivalent to that in the training session (the difference was −1.07%; see Fig. 5c), *t*(29) = −0.304, *p* = .76, *BF*_*10*_ = .203. In other words, contextual facilitation acquired with low-contrast displays remained unchanged when switching from mesopic to photopic vision.

### Discussion

In line with Experiment 2, Experiment 3 confirmed that invariant contexts, whether encountered under high or low stimulus-contrast conditions, could be effectively acquired and retrieved in mesopic vision. In addition, the results of Experiment 3 showed that learned contexts can be transferred from dark mesopic to bright photopic environments when display contrast remains high. However, the contextual-cueing effect was rendered statistically marginal when the display contrast remained low after the switch to photopic vision.

## Recognition test

All participants were administered a recognition test after the visual-search (training and test) sessions of each experiment. Prior to the recognition test, participants were explicitly told that they would be presented with equal numbers of old and new displays. Given this information, participants who provided the same, biased response (e.g., responding consistently “new” or “old”) throughout the entire recognition session were excluded from further analysis. Altogether, three participants from Experiment 2B and one participant from Experiment 3A were excluded.

**Fig. 6:**
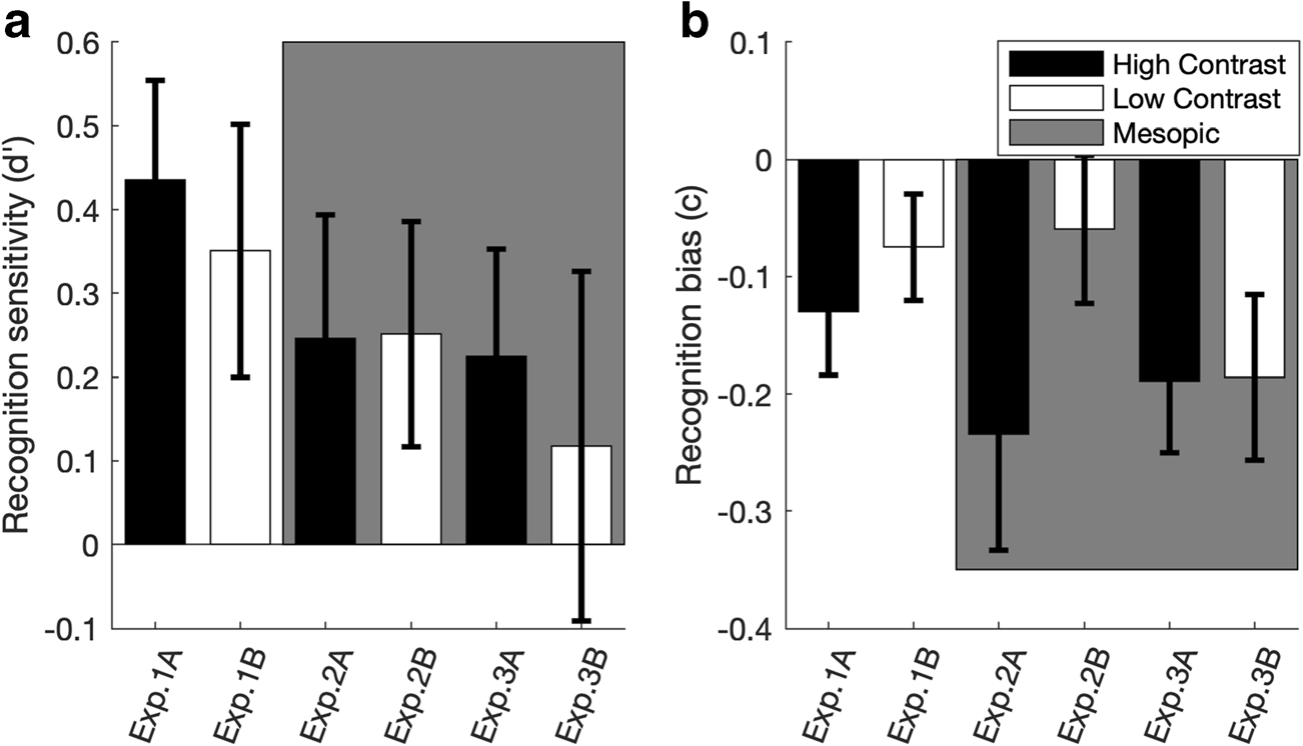
**a** Mean recognition sensitivity (*d'*), and (**b**) mean response bias *c*, for all experiments. The error bars indicate one standard error. Gray background shading denotes that the recognition test was conducted in mesopic vision; white background that it was run in photopic vision

For the remaining data, participants’ mean hit and false-alarm rates were calculated, which turned out as follows: Experiment 1A, 62.5% versus 46.67%, *t*(29) = 3.67, *p* = .001, Cohen’s *d* = 0.37; Experiment 1B, 58.8% versus 46.3%, *t*(29) = 2.29, *p* = .03, Cohen’s *d* = 0.42; Experiment 2A, 60.0% versus 54.2%, *t*(29) = 1.41, *p* = .17, Cohen’s *d* = 0.26, *BF*_*10*_ = 0.47; Experiment 2B, 56.5% versus 47.7%, *t*(27) = 1.78, *p* = .087, *Cohen’s d* = 0.34, *BF*_*10*_ = 0.81; Experiment 3A, 61.2% versus 52.6%, *t*(28) = 1.93, *p* = .064, Cohen’s *d* = 0.36, *BF*_*10*_ = 0.99; Experiment 3B, 56.2% versus 55.0%, *t*(29) = 0.23, *p* = .82, Cohen’s *d* = 0.04, *BF*_*10*_ = 0.20. We further examined the old-/new-display discrimination sensitivities (*d'*) and the associated response biases (*c*) per experiment (see Fig. 6 for the results). One-sample *t* tests revealed the *d'* score to be significantly greater than zero in the photopic vision condition: Experiment 1A, *t*(29) = 3.66, *p* = .001, mean effect of .44; Experiment 1B, *t*(29) = 2.32, *p* = .027, mean effect of .35, but not in the mesopic condition (Experiments 2–3; all *t*s < 1.88, all *p*s > .05, *BF*_*10*_ < .94), providing some evidence of explicit learning under photopic conditions (in Experiments 1A and 1B), consistent with the finding of previous studies (Schlagbauer, Müller, Zehetleitner, & Geyer, 2012; Smyth & Shanks, 2008). By contrast, there was no evidence of explicit learning under mesopic conditions. However, a cross-experimental comparison (one-way ANOVA with the between-subject factor lighting environment) failed to reveal the difference in *d'* between the mesopic and photopic conditions to be significant (while also not providing strong support for a null effect): 0.21 versus 0.39, *F*(1, 174) = 2.02, *p* = .157, *BF*_*10*_ = .435. Thus, from the present data, there is insufficient evidence to firmly conclude that contextual learning involves a greater degree of “explicitness” under photopic as compared with mesopic vision conditions.

A second group of *t* tests of the response-bias scores *c* (Fig. 6b) reached significance in Experiments 1A, 2, 3A, and 3B (*t*s < −2.3, *p*s < .026, *BF*_*10*_ > 2.03), but not in Experiments 1B and 2B (*t*s > −1.7, *p*s > .11, *BF*_*10*_ < .65). There is no clear pattern in the bias scores across the six subexperiments, except that, in general, participants tended to respond rather conservatively (i.e., they tried to avoid false “old” responses) in the recognition test (as indicated by the negative *c* values).

## General discussion

The present study investigated the acquisition of spatial context cues and their retrieval with high-contrast and low-contrast displays under photopic and mesopic ambient-light conditions. The results revealed differential effects of luminance contrast on contextual learning and retrieval. Contextual cueing was effective with high-contrast displays, but was multifaceted with low-contrast displays. Low-contrast contexts could be learned under both mesopic and photopic vision, but were retrievable only under mesopic conditions. Learned context cues were transferred from low-contrast to high-contrast conditions, but not vice versa. When the environmental lighting was switched from mesopic to photopic, retrieval of low-contrast contexts was only marginally significant. Taken together, our findings suggest that low-contrast contexts can be used much better in mesopic than in photopic vision; and display contrast plays a critical role for contextual retrieval in photopic vision.

### Display contrast and contextual cueing

The finding that contextual retrieval works only with high-contrast (but not low-contrast) displays in the photopic environment is theoretically important, given that contextual cueing is thought to be a robust phenomenon, manifesting in various complex environments (Goujon et al., 2015). For instance, contextual cueing has been reported in search tasks with multiple redundant contexts (e.g., scene-based plus space-based contexts in Brooks et al., 2010), across different types of sensory modalities (e.g., tactile modality in Assumpção, Shi, Zang, Müller, & Geyer, 2015, 2018; and auditory modality in Kawahara, 2007), and even when only part of the context in the search arrays remained constant (Brady & Chun, 2007; Jiang & Chun, 2001). It should be noted that, in most of the previous studies, (visual) contextual-cueing effects were observed with high-contrast configurations. However, our results revealed that stimulus contrast is a critical factor for facilitation to become manifest under conditions of photopic vision: low-contrast displays increase the difficulty of discriminating the search items from the background as well as discriminating the target from the distractor items—in particular, as concerns items in peripheral vision—hampering participants to apprehend or retrieve the spatial inter-item relations. As a result, even with 25 blocks of repetition, which typically suffice to engender robust contextual cueing (e.g., Annac et al., 2013; Zang, Geyer, Assumpção, Müller, & Shi, 2016; Zellin et al., 2013), we failed to observe any significant contextual cueing for the low-contrast display in photopic environment (in Experiment 1B).

The contrast dependence of contextual retrieval is consistent with accounts according to which the luminance contrast alters the size of the “visual span”—the region of the visual field from which information can be effectively taken up within an eye fixation (Hulleman & Olivers, 2017; Rayner, 1998; Shi, Zang, & Geyer, 2017). For instance, Näsänen et al. (2001) had participants search for an uppercase letter in arrays of numerals; they found the visual span to become smaller as the contrast decreased, evidenced by reduced saccade amplitudes and increased fixation durations. Contraction of the perceptual span means that only a reduced number of search items can be processed per fixation, likely impeding the retrieval of the spatial inter-item relations that are necessary for contextual cueing to manifest. Consistent with this, Zang et al. (2015) found contextual cueing to be diminished when the visual span was limited to two to three items by making participants search the displays under conditions of gaze-contingent “tunnel vision”; see also Geringswald and Pollmann (2015), who found that contextual cueing was completely abolished both in a training session with loss of peripheral vision with induced by tunnel vision and a subsequent transfer session without tunnel vision. In the relevant experiments of the present study, changing the contrast from high to low likely brought about a contraction of the visual span, impeding the retrieval of learned context cues—which is supported by the fact that the pattern we observed in Experiments 1A and 2A is very similar to our previous findings with gaze-contingent display presentation (Zang et al., 2015).

It should be noted that low stimulus contrast may not impede contextual learning. In the photopic environment (Experiment 1B), although contextual cueing was not significant with the low-contrast displays in the training session, contextual facilitation manifested already in the first block of the transfer session with the high-contrast displays, suggesting that contextual learning did take place in the preceding training session. In other words, in the photopic environment, low-contrast displays allow for contextual learning, while affecting the expression of contextual cueing. This is similar to the findings with gaze-contingent displays with a restricted foveal view of two to three items (Zang et al., 2015): Contextual learning remained intact, but contextual retrieval was compromised. Collectively, these findings suggest that configural relations are constructed from (near-)foveal information acquired across saccades. In each fixation, local items and their relative positions are analyzed and, once the target is detected, the configural context of the items scanned during the last few fixations is buffered in a visuo-spatial short-term memory for (saccadic) search guidance, for instance, in form of the “scanpath” traversed (see also Guang, Liu, Jiao, Zhou, Li, Sun, & Zhao, 2012; Manginelli & Pollmann, 2009; Zang et al., 2015). From these representations, long-term learning of contextual regularities becomes possible (at least to some extent), even when the uptake of information from peripheral vision within a given fixation is (severely) restricted. By contrast, contextual retrieval from long-term memory involves a template-matching process, which requires uptake of some more global visuospatial information (from a wider region around fixation) for the appropriate context-memory template to be triggered and cue search to the target location. This could explain why contextual cueing was expressed in the transfer session of Experiment 1B, after display contrast was switched from low to high: The switch made more peripheral information available, facilitating template matching and contextual search guidance.

### Lighting condition and contextual learning and retrieval

Changes of the activation ratio between the cone and rod systems and, as a result, of the visual span are likely to alter the search strategies from photopic to mesopic vision. The activation of the peripheral rod system in mesopic/scotopic vision may compensate for the limited availability of information from the foveal region under conditions of low display contrast. This receives support from our observation that contextual learning and retrieval function relatively independently of display contrast in mesopic environments (as long as enough training, e.g., over five epochs, is provided). When performing search in photopic vision, observers tend to shift attention overtly to different locations in the search display by making saccades and then focus processing on the immediate region represented within and around the fovea to find the target (Paulun et al., 2015). This more “local,” fovea-centered search strategy yields higher detection probabilities for targets located closer to the fixation position, especially in the display center, while more saccades need to be made to find a target located in the periphery. By contrast, when searching under conditions of scotopic vision (under which foveal stimuli are degraded), fixation durations increase compared with photopic conditions (Paulun et al., 2015), reflecting both the delayed information processing in the rod pathway (Zele et al., 2013) and more “global” scanning, within an eye fixation, over an extended peripheral range to find the target (Paulun et al., 2015).

These differential search modes—more fovea-centered, local search in photopic vision versus more peripheral, global search in mesopic vision—are likely to be the main cause of the differential contextual-cueing effects with low-contrast displays between photopic (no significant cueing in Experiment 1B) and mesopic vision (significant cueing in Experiment 2B). Previous studies have shown that the availability of the global spatial configuration plays an important role in contextual learning and retrieval (Beesley, Vadillo, Pearson, & Shanks, 2015; Higuchi & Saiki, 2017; Zang et al., 2015). For instance, Beesley et al. (2015) showed that preexposing observers to the global distractor configuration (with targets positioned pseudorandomly across trials) in an experimental phase prior to a phase with consistently placed targets within the global configuration can lead to more efficient search guidance in the second phase. Similarly, Zang et al. (2015) found that trial-wise providing observers with a brief preview of the whole configuration in a placeholder display (of crosses) prior to gaze-contingent scanning of the search display (of *L*s and a *T*) can aid contextual retrieval. With regard to the present study, we propose that in mesopic vision, the effective contextual learning and retrieval of low-contrast contexts is attributable to the more global search mode supported by the (peripheral) rod system. In photopic vision, by contrast, contextual retrieval was likely hampered by the more local search mode induced by low-contrast displays. Interestingly, repeated contexts could still be learned in local search mode, as evidenced by the significant cueing effect manifesting already in the first block of the transfer session, after the switch of the display contrast switched from low to high. This is consistent with previous findings (Annac, Conci, Müller, & Geyer, 2017; Zang et al., 2015) emphasizing the role of local target–distractor associations in contextual learning.

### Variability of context and visual span in contextual learning

One might argue that the present findings, rather than arising from systematic variation of the visual span brought about by the changes in ambient lighting and stimulus contrast, just reflect the variability of the available contextual information in different conditions. That is, low-contrast (vs. high-contrast) displays cause more variability in the learning (e.g., Experiment 1B) and retrieval phases (Experiments 1A and 2A), rendering contextual-cueing effects nonsignificant; similarly, environmental changes introduce variability in the availability of context cues (e.g., Experiment 3B), rendering the cueing effect statistically “marginal.” However, while the variability account provides an explanation of the effect pattern on an information-processing level,^1^ it tells us little about the functional level. The visual-span account, by contrast, specifies functionally what information is taken up under which conditions (e.g., fovea-centered local vs. peripheral global mode of processing) and why (balance of rod and cone systems, adaptation of the visual span)—that is, it offers an explanation why the available contextual information may be more variable under some compared with other conditions. Of course, the accounts at the two levels may not map one-to-one onto each other—for instance, it is conceivable that the visual span remains the same across different lighting and contrast conditions, but the rate of information accumulation differs among these conditions. Based on the (manual-RT) data collected in the present study, such conditions cannot be discerned. Future studies involving manipulation of the display complexity and combined recording of eye movements and manual RTs may help us dissociate these two accounts.

### Implicit versus explicit memory in contextual cueing

What do our findings tell with regard to the question of whether contextual cueing is based on an explicit or an implicit memory system? It is important to note that explicit knowledge of repeated displays does not guarantee a significant contextual-cueing effect (see Results of Experiments 1B and 2B), suggesting that the cueing effects in visual search task and observers’ ability to explicitly discriminate repeated from non-repeated displays may be decoupled. It has long been debated and remains controversial whether automatic contextual guidance and conscious recognition are based on a single memory system that operates in a two-stage process (e.g., Annac et al., 2019; Kroell, Schlagbauer, Zinchenko, Müller, & Geyer, 2019), or a dual-memory system with separable, implicit and explicit processes (e.g., Colagiuri & Livesey, 2016). For instance, Annac et al. (2019) have recently advocated a single-memory account, according to which context-based guidance of visual search is mediated by a fast, nonconscious process at a first processing stage, while conscious recognition is the outcome of a slower-operating, second stage in which (aided by local, fovea-based information) the output of the first stage is rendered consciously accessible. By contrast, Colagiuri and Livesey (2016) have argued that context-based facilitation is supported by an implicit memory system and recognition by a separate, explicit system. Of note, however, the single-systems versus dual-systems debate has been primarily based on findings from near-photopic laboratory environments. The present findings suggest that different lighting environments may influence observers’ search mode (more global vs. more local), which could have differential impacts on the search and recognition tasks employed in contextual-cueing studies. However, in light of the limited power of our recognition test (Colagiuri & Livesey, 2016; Vadillo, Konstantinidis, & Shanks, 2016), this proposal—which rests on a null result (no significant explicit recognition in mesopic vision, though without recognition performance being significantly lower compared with photopic vision)—must be interpreted with caution. In any case, further work under differential environmental lighting conditions is necessary to corroborate this idea.

### Array search and scene search

Would our findings obtained with meaningless artificial displays generalize to search in meaningful, naturalistic scenes? As noted briefly in the Introduction, guidance of visual search in naturalistic scenes involves additional cues deriving from “scene grammar” (Võ & Wolfe, 2013; Wolfe et al., 2011)—that is, certain objects are congruent (others incongruent) with a scene’s meaning (semantic consistency), and objects do (or do not) comply with structural rules (syntactic consistency). For example, the presence of a computer mouse (but not that of a bar of soap) would be semantically consistent with a desktop scene containing a computer; and a mouse located next to the computer (but not one positioned on the screen) would be syntactically consistent with the scene (see also Torralba, Oliva, Castelhano, & Henderson, 2006; Vo & Henderson, 2009; Wolfe et al., 2011). Accordingly, search may be guided by knowledge of the prior probability of the presence and the location of an object within a scene, and this knowledge may be rapidly activated by recognition of the essential (low-level) “gist” of a scene (e.g., Bahle, Matsukura, & Hollingworth, 2018; Fei-Fei, Iyer, Koch, & Perona, 2007). However, while the gist of a scene provides us with an idea of what the scene is about (semantically), which essential components to expect, and how these are structurally organized, individual “target” objects may have a more or less “arbitrary” relation to the scene (e.g., a kettle may not be part of each kitchen scene) and they may still occupy locations that can vary widely (e.g., in a kitchen scene, the kettle is likely to be found on a flat surface at half height relative to the ground, but it can be located on any such surface, and anywhere on such a surface). Thus, while search would be constrained, or guided, by the overall-scene gist, there would still be ample scope for (both more global and more local) contextual learning. Although in the traditional contextual-cueing paradigm, the search arrays themselves are meaningless, and there is no semantic, “gist”-based guidance, we believe it can still serve as a useful model for studying the acquisition of arbitrary contextual inter-item relations (with implications even for natural scenes). Given that changes of the lighting and stimulus contrast conditions engender similar adjustments of basic-level visual information processing (balance of the rod/cone system, adjustments of the perceptual span) regardless of whether the scene is artificial or naturalistic, we would expect the current findings to largely extend to natural scenes, too. In reading, for instance, semantic word predictability may enlarge the perceptual span (Hohenstein & Kliegl, 2014; Rayner, 2009),^2^ and this may conceivably also be the case with the predictions afforded by scene-grammar cues—compensating, to some extent, for low-level perceptual constraints. Establishing this is an empirical matter, which is beyond the scope of the current study.

### Limitations and outlook

It should be noted that the focus of the current study was on contextual learning in mesopic vision and transfer to photopic vision. A key new finding was that activation of the (peripheral) rod system in mesopic vision may help both the learning and the retrieval of low-contrast contexts. One possible limitation of the present study, though, is that we tested (only) six combinations of luminance contrast and environmental lighting in the training and test sessions, leaving other potentially important conditions untested, such as contextual learning in the photopic environment and retrieval in the mesopic environment while fixing the display contrast at either low or high. Yet the outcomes from the combinations that we tested allow us to predict, with confidence, what would happen under these conditions. Specifically, contexts encountered at high stimulus contrast can be successfully learned (Experiments 1A, 2A, and 3A) and retrieved (Experiments 2B and 3A) not only in photopic environments (consistent with a great many published contextual-cueing studies; see Goujon et al., 2015, for a review), but also in mesopic environments. Consequently, for high-contrast displays (in both the training and transfer sessions), contextual cueing is highly likely to transfer from photopic to mesopic vision. With low stimulus contrast, we did not observe reliable contextual cueing in the photopic environment (Experiment 1B). Accordingly, if contrast stays low, we would not expect to see any transfer of cueing with a change from photopic to mesopic environments.

It would, of course, be of interest, in future work, to investigate other combinations of luminance contrast and environmental lighting, such as contextual learning of high-contrast contexts in photopic vision and testing transfer to low display contrast in mesopic vision. The pattern of transfer effects would help us better understand whether the activation of the peripheral rod system in mesopic vision facilitates contextual retrieval processes.

### Conclusion

In summary, the present study, for the first time, examined the effects of display contrast and ambient lighting on contextual learning and retrieval. The results revealed increasing luminance contrast to boost contextual cueing in general, likely by allowing search to operate with an increased perceptual span. Importantly, whereas contextual retrieval (but not necessarily learning) is compromised with low-contrast displays under photopic conditions, it is effective under mesopic conditions. These differential effects are likely attributable to differential search modes in mesopic versus photopic environments: A more local, fovea-centered mode in photopic vision hampers contextual retrieval with low-contrast stimuli, but helps explicit recognition of repeated displays; in contrast, a more global search mode in mesopic vision aids contextual cueing, but may impede explicit recognition. Thus, effective contextual learning and retrieval depend on the search mode induced by the ambient lighting, and specifically a global mode mediated by the rod system coming into play under conditions of mesopic vision. Concerning the latter, our finding of successful contextual learning and transfer from low to high display contrast in the mesopic environment may have interesting implications for contextual learning in real-life (applied) scenarios. For example, complex road layouts (for navigation in both night and daylight conditions) may actually be more efficiently learned if these are encountered under low-luminance night-vision conditions first.

#### Author note

This research is supported by the cultivation project of the province-leveled preponderant characteristic discipline in the college of education of Hangzhou Normal University (18JYXK027) and , the Starting Research Fund from Hangzhou Normal University (RWSK20190315), the National Natural Science Foundation of China (NSFC 31600876) to X.Z., and the German Research Foundation (DFG, grants SH 166/3-2) to Z.S. The funding bodies play no role in the study design, data collection, analysis, decision to publish, or preparation of the manuscript.

#### Open practices

The data and codes for all experiments are available (https://github.com/msenselab/mesopic_contextual_cueing).

## Footnotes

1 Arguably, though, this is a post hoc explanation. It may be hard to predict a priori under which conditions there is greater variability in the availability of contextual cues.

2 Note, though, that this is not universally accepted.
